# Women’s experiences of sexuality during menopausal transition: An interview study

**DOI:** 10.1016/j.eurox.2026.100462

**Published:** 2026-04-25

**Authors:** My Högblom, Anette Lycke, Karin Fristedt, Ewa Andersson

**Affiliations:** a Lund University, Centre for Primary Health Care Research, Department of Clinical Sciences, Sweden; b Karolinska Institute, Women’s and Children’s Health, Sweden

**Keywords:** Experiences, Menopausal transition, Sexuality, Women, Qualitative study

## Abstract

**Objective:**

Menopausal transition, brings significant changes to various aspects of health and well-being, including sexuality. These changes are influenced by hormonal changes, psychological factors, and societal perceptions. Historically, research on menopausal transition has predominantly focused on pharmacological treatments, to address symptoms. However, these studies often neglect the inclusion of women’s personal experiences and perspectives on sexuality. This study aimed to explore women’s lived experiences of sexuality during menopausal transition.

**Methods:**

Data were collected by using semi-structured individual interviews with ten women aged between 50 and 60. The data was analysed using Malterud systematic text condensation.

**Results:**

The women reported shifts in sexual desire and physical intimacy during menopausal transition, and they discussed their evolving perceptions of sexuality and its role in their lives. The women emphasized that the quality of their relationship and partner-related sexual dysfunction were important factors influencing their sexual experiences. Finally, participants noted that the limited discourse surrounding sexuality during the menopausal transition contributes significantly to a broader lack of awareness and inadequate understanding of women’s sexual health in this life stage.

**Conclusion:**

Women's sexuality during menopause is influenced by both biological and psychosocial factors, and support from healthcare services is often lacking. Communication within the couple is crucial, and men's sexual health and experiences are also important. More individualized, culturally sensitive, and inclusive care, as well as sexual counselling, can improve couples’ well-being and quality of life during this stage of life.

## Highlights

• First Swedish study on women’s perceptions of sexuality during menopause.
• Women lack awareness of how sexuality impacts their well-being.
• Women report experiencing sexual desire, but relationship issues act as a barrier.

## Introduction

Menopause marks a natural transition experienced by all women, yet it continues to be surrounding by cultural taboo and is often primarily associated with ageing and the cessation of sexual activity. The menopausal transition typically spans 5–10 years before and after the final menstruation, with the median age of onset being approximately 52 years [1]. Clinically, menopause is confirmed after 12 consecutive months without menstruation. Following this transition, oestrogen levels continue to decline for 4–5 years, frequently giving rise to physical symptoms such as hot flushes, vaginal dryness, night sweats, and palpitations [2]. These physical changes, along with psychological stress and broader health concerns, can have a significant impact on women’s overall well-being during menopausal transition.

Sexual health, an integral component of overall health, is defined not solely by the absence of disease or dysfunction, but also by the presence of physical, emotional, mental, and relational well-being [3]. For many women, menopausal transition brings physical and hormonal changes that may compromise sexual health, with many women reporting declines in sexual desire and arousal, difficulties with orgasm, and pain during sexual activity 2005; [2]). Psychosocial stressors, including sleep disturbances, caregiving responsibilities, and the transition of children leaving home, may further disrupt sexual well-being [4].

Evidence demonstrates a gradual decline in sexual function and frequency across midlife, which is exacerbated by the physiological and hormonal alterations characteristic of the menopausal transition [5]. This decline has been associated with diminished quality of life, emotional distress, and social isolation [6]. While sexual dysfunction during menopause is not classified as a disease, it reflects a complex interplay between somatic and psychological responses to hormonal change. The existing literature has predominantly focused on biomedical explanations, particularly the effects of declining oestrogen on desire, arousal, and vaginal lubrication [2].

Epidemiological studies highlight the scope of this issue, showing that sexual disorders increase from 27.2% among women aged (18-44) to 44,6% among those aged 45–64 [7]. Declining oestrogen contributes to physiological changes in the vaginal wall, such as thinning of the lining, which can result in reduced lubrication and painful intercourse (dyspareunia) [8]. Hypoactive sexual desire disorder (HSDD) is the most common sexual complaint among postmenopausal women, characterised by a persistent lack of desire, fantasies, or interest in sexual activity for at least six months, often accompanied by personal distress or relational strain [8], [9].

Although therapeutic models, such as sexological therapy, have been available since the 1960s, research remains limited on strategies to maintain or enhance sexual desire during menopause. Clinical frameworks – such as Basson’s Motivational Sexual Response Cycle, which addresses women’s sexual response, and the PLISSIT model – underscore the importance of emotional intimacy and structured therapeutic guidance in addressing sexuality in midlife [10].

Moreover, recent findings suggest that women’s concerns also extend to their partners’ satisfaction, adding further complexity to the dynamics of sexual well-being during this life stage [11].

Despite these insights, relatively little is known about how women themselves perceive and navigate the sexual changes associated with menopause. This study therefore explores women’s lived experiences of sexuality during the menopausal transition, paying particular attention to the emotional, relational, and social determinants of sexual well-being.

## Methods

### Design

A qualitative interview methodology was employed in this study, with data analysis guided by Systematic Text Condensation (STC), as outlined by Malterud [12]. This analytical approach is rooted in Giorgi’s phenomenological method [13], providing a structured approach to capturing participants’ lived experiences.

### Sampling and recruitment

Participants were recruited using a combination of convenience and snowball sampling [14]). Initial participants were identified through the research team’s professional and personal networks and through announcements posted on social media platforms. Any pre-existing relationships with participants were identified and carefully managed to minimise undue influence.

After enrolment, participants were invited to refer one additional eligible acquaintance. To minimise clustering within the same social networks and reduce potential homogeneity in the sample, each participant was limited to inviting only one additional individual. Eligibility criteria included being genetically female, 50–60 years of age, and proficient in Swedish. Prior to participation, all individuals were sent information and a consent form via email. The consent form was signed and returned electronically by all participants before the interview. Additionally, verbal consent was recorded at the beginning of each interview.

### Data collection

The authors developed a semi-structured interview guide, which included both closed and open-ended questions to allow for a transition to more exploratory inquiries during the later stages of the interview. Individual interviews were conducted with each participant between September and October 2024, with two researchers present: one leading the interview and the other observing and taking notes to enhance data quality, minimise bias, and allow immediate reflection. All authors had prior training and experience in qualitative interviewing and were familiar with semi-structured methods, ensuring consistency and rigour in data collection.

The interviews were carried out through video meetings on Microsoft Teams or via telephone, following the participants’ informed consent. Their duration ranged from 30 to 60 min. All the interviews were recorded and subsequently transcribed using transcription software, followed by manual verification by the authors to ensure accuracy. All quotes included in this article were translated into English by the authors.

A pilot interview was conducted to assess the clarity, relevance, and comprehensibility of the questions in the interview guide, resulting in minor wording changes to enhance clarity. Additionally, the pilot participant was asked to evaluate whether the interview content aligned with the expectations outlined in the study’s previously communicated aims.

### Analysis

The interviews were analysed using Malterud’s [12] Systematic Text Condensation (STC), a four-step version of thematic analysis that ensures a systematic and transparent qualitative interpretation. Firstly, two authors independently conducted a preliminary reading, during which they identified eight initial themes. These gave an early impression of recurring topics or emotional undercurrents in the data. Their purpose was to guide attention during coding and to serve as a reference point for the evolution of final themes. These preliminary themes were later revisited, merged, refined, or discarded depending on how the data developed, which guided subsequent coding. Secondly, meaning units relevant to the study aims were extracted and coded, with codes clustered to reveal cross-case patterns. Thirdly, these codes were condensed into subgroups reflecting shared meanings across participants, enabling nuanced insights.

Final themes were generated by identifying overarching concepts that captured the essence of multiple related categories, emphasising both commonalities and nuanced variations in participants’ accounts. Throughout, collaborative discussions and reflexive practices ensured that potential biases were continuously interrogated, maintaining a thematic structure grounded in participants’ perspectives rather than researchers’ assumptions. Researcher reflexivity and positionality were considered throughout the study, including reflections upon professional backgrounds, prior assumptions, and the use of reflexive practices such as team discussions and analytical memos.

This iterative process led to the identification of four overarching themes, each supported by several categories derived from multiple codes and meaning units.

For examples of the process of text condensation, see Table 1.

**Table 1:** Example of analysis.

Preliminary themes	Meaning units	Codes	Categories	Themes
Attitudes towards talking about sex and the menopause	It’s taboo to talk about, depending on which generation you belong to	Taboo to talk about sex	Desire for increased access to advice and information	Perceived understanding and lack of awareness
Sex during menopause	…it affects the relationship that I’m not turned on all the time	Displeasure can affect relationship negatively	The importance of communication	The importance of relationships
Factors that influence sex drive	The sex drive goes down – it really has a lot to do with how tired you get when you don’t sleep well. It affects you a lot	The impact of sleep on sexuality and sex drive	Identified barriers to sexual intimacy	Reflections upon sexuality

**Table 2:** Overview of themes and categories.

**Themes**	**Categories**
**Changes affect sex life**	**Causes and symptoms that affect sex drive**
	**Concurrent circumstances affecting sex drive**
	**Confidence in yourself and your own body during menopause**
**Reflections upon sexuality**	**Expectations of sexual activity**
	**Reflections upon a fulfilling sex life**
	**Identified barriers to sexual intimacy**
**The importance of relationships**	**The importance of effective communication**
	**Absence of sexual intimacy and disappointment**
**Perceived understanding and lack of awareness**	**Perceptions of menopausal treatment**
	**Access to advice and information**

### Ethics

Prior to participation, all individuals were emailed a consent form outlining the details of the study. This document, which participants signed before the commencement of the interviews, emphasised the voluntary nature of their involvement. It explicitly stated that participants could withdraw from the study at any point without providing a reason. Furthermore, the consent form assured participants of the confidentiality and anonymity of their sociodemographic data, and any materials collected. The study was approved by the Regional Ethical Review Board (Dnr: 2024–02583–01).

## Results

### Participants

The study sample comprised ten semi-structured interviews with women aged 50–60, residing in different regions of Sweden and representing a range of occupational sectors. All participants had personal experience of the menopausal transition. Those in relationships were living with male partners, while one participant was single.

### Changes affect sex life

This theme illustrates the ways in which women’s sex lives were influenced by multiple, intersecting factors during menopause. Physical and psychological symptoms, daily life circumstances, and perceptions of the body all shaped sexual desire and intimacy.

#### Causes and symptoms that affect sex drive

Both physical and psychological menopausal symptoms were reported to have an impact on women’s sexual experiences. Common issues included fatigue, hot flushes, vaginal dryness, dyspareunia, mood swings, bodily changes, and cognitive difficulties. The duration and intensity of symptoms varied widely, from brief and mild to prolonged and disruptive.

The women described the factors contributing to reduced sexual desire as being linked to a gradual decline over time. They reported a noticeable reduction in sexual excitement, which they primarily attributed to hormonal fluctuations. Optimal external conditions and overall well-being were identified as key factors in enhancing sexual desire. While some participants reported an increase in sexual desire following the onset of menopause, others noted no significant change. In evaluating their motivation to initiate sexual activity, they consistently rated psychological desire as more prominent than physical arousal. One participant provided a perspective on this subject.

*We just needed some help sometimes, simply because it gets dry, that’s just how it is, so I noticed that. But it wasn’t that I was thinking about it. I didn’t have any itching or anything like that, it was just in those, …in those situations that I noticed it, so to speak*. (Participant 7)

#### Concurrent circumstances affecting sex drive

Participants described occupational demands, fatigue, and longer recovery times with age as key factors diminishing sexual desire. They further highlighted that sexual activity was shaped by stress, family health challenges, the continued presence of adult children in the household, and the physical changes accompanying menopausal transition. Energy levels played a crucial role in facilitating desire, enabling greater bodily responsiveness. The women also emphasised the importance of actively nurturing desire, contributing to a positive cycle of improved sexual well-being. Distinguishing menopause-related changes from factors such as illness, stress, remote working, weight gain, and the impact of COVID-19 in the past was challenging. Participants suggested that setting aside dedicated time for intimacy could be an effective strategy for enhancing sexual activity.

The decline in sexual vitality was often attributed to various factors, including limited time, a mutual lack of enthusiasm in initiating desire, and reduced sexual interest from their partner, as one participant explained:

*I would say that I have a bit less sex than I would like. And that’s why I’m bringing it up, that I really need to address this issue with my husband and genuinely bring it into the light.* (Participant 8)

#### Confidence in yourself and your own body during menopause

Trusting the body’s natural processes during menopause supported a sense of security in one’s biological femininity. Embracing these physical changes emerged as a vital factor for mental well-being, encouraging feelings of maturity and comfort within one’s body. The capacity to attune and respond to bodily needs, as well as to experience pleasure, was strongly tied to self-esteem.

A prevailing sentiment among participants was that increased self-assurance led to a greater emphasis on self-care, which became essential not only for overall well-being but also for sexual health. This perspective is encapsulated in the following quote: ‘*I’m more forgiving of my body because it will last a few more years, and I want to take care of it*’ (Participant 10).

### Reflections upon sexuality

This theme highlights the ways in which participants reflected upon the meaning and role of sexuality during the menopausal transition. Their accounts show how expectations, the pursuit of fulfilment, and the experience of barriers shaped their sexual well-being and intimate relationships.

#### Expectations of sexual activity

The participants viewed sexual activity as an important part of life, focusing more on the quality of their sexual experiences than on frequency. Although definitions of sex varied among the women, most did not regard penetrative intercourse as essential. However, for some participants, achieving orgasm was consider particularly significant.

A common feeling among the women was the desire for a more spontaneous sex drive, similar to what they had experienced in their younger years. They also recognised the need to build greater confidence and skill in initiating sexual activity, whether with a partner or through self-stimulation, as one woman explained: ‘*now you have to decide to do it, and really, there are only opportunities*’ (Participant 1).

As the women aged, they generally felt that their sex lives had improved. Increased self-esteem enabled them to feel more comfortable expressing their sexual desires to their partners, which contributed to a more fulfilling and emotionally enriching sexual experience. An expectation among the women was that sex should be a natural part of life, comparable to eating and exercising, and they viewed it as essential to both physical and mental well-being.

*Pleasure itself is a joy. The body feels good, the mind feels good, so it’s one of the most important things we have.* (Participant 8)

Throughout the interviews, a potential strategy for enhancing sexual activity was identified as allocating more time and prioritising sexual intimacy. Participants frequently attributed their decline in sexual vitality to several factors, including time constraints, a mutual lack of enthusiasm in initiating desire, and a partner’s diminished interest in sexual engagement.

#### Reflections upon a fulfilling sex life

The women regarded a satisfying sex life as one that encompasses both pleasure and emotional intimacy with their partners, recognising it as a vital aspect of life deserving of greater attention. They emphasised that sexual satisfaction contributes significantly to overall well-being and life satisfaction. The following quote illustrates the importance of sex for different participants: ‘*Sex makes you happier and you feel more uplifted. Sometimes you wonder if you become a bit depressed when it disappears*’ (Participant 9).

For those no longer in a sexual relationship with their partner, masturbation played a crucial role in their sexual satisfaction and pleasure. It served as a form of self-exploration, enabling women to become more familiar with their bodies while also preserving a sense of sexual vitality, independent of a partner.

*Being able to have an orgasm is something that, for some reason, comes up if I start to fully focus on myself, and what I think is connected to feeling alive. That’s what I believe. Alive in relation to, in contrast to, feeling like you’re dying.* (Participant 6)

#### Identified barriers to sexual intimacy

The cessation of sexual activity reported by participants was frequently attributed to erectile dysfunction or a lack of sexual desire in their male partners, often resulting from an underlying medical condition or medication that affected erectile function. Additionally, there was a sense of resignation regarding the belief that influencing sexual desire felt unattainable, as articulated by one of the women.

*I can’t change anything to influence my sex drive. I’ve tried things that people just talk about. Magnesium is one thing that’s supposed to increase sex drive, but it doesn’t, and I have to accept that I’m in menopause and I have no sex drive; the biological clock has stopped, as they say. I believe that’s what it is*. (Participant 2)

In cases where women were cohabiting with partners experiencing sexual dysfunction, it was observed that the partners chose not to seek treatment. This reluctance to pursue intervention contributed to a persistent sense of unspoken distress among the women, ultimately leading to the absence of a mutual sexual relationship. Participants reported that this situation led to emotional isolation and dissatisfaction, significantly reducing their overall quality of life and intimate connections.

*Even though it’s a difficult question for him. Because in the end, it affects me negatively. I start to believe that there’s something wrong with me, and I know what it’s about.* (Participant 8)

The decline in sexual desire, whether in the woman or her partner, led to a significant reduction in instances of sexual intimacy. Furthermore, the use of sex aids, such as lubricants, was often viewed negatively and considered a ‘turn-off’, particularly in the context of decreasing frequency of sexual encounters.

### The importance of relationships

Participants highlighted that strong relationships rely on communication and sexual intimacy. Clear communication fosters connection, while its absence makes expressing needs difficult. Sexual intimacy acts as a ‘glue’, and without it, relationships can drift towards emotional distance or friendship-like bonds.

#### The importance of effective communication

Establishing effective communication with one’s partner was recognised as essential for maintaining a respectful and supportive relationship. Non-verbal communication, shared values, and the connection fostered through mutual laughter were highlighted as essential components of a fulfilling relationship. Conversely, when communication broke down, women who refrained from sexual activity with their partners often found it difficult to articulate their desires and sexual needs. This reluctance was frequently driven by a fear of assigning blame or negatively influencing her partner’s self-esteem.

*What decreases my sex drive is when someone withdraws, I find that difficult. Don’t expect me to sense the atmosphere, because then I start to believe there’s something wrong with me.* (Participant 8)

#### Absence of sexual intimacy and disappointment

The interviews revealed that the women had not initially considered the absence of sex or its implications until prompted. Upon reflection, they acknowledged that they had consciously prioritised other aspects of life over maintaining an active sex life. This realisation encouraged deeper introspection, often accompanied by feelings of sadness and aggression.

*When you don’t have sex often, I think it’s easier to drift apart, and it’s almost easier to get angry at each other. In fact, I think you lose that closeness, which is so important. It’s something like a kind of glue in the relationship that I think helps. You feel like you’re one unit then*. (Participant 4)

When asked about sexual inactivity, the women reflected upon their once-fulfilling sex lives, contrasting these memories with a current sense of loss. The absence of sexual intimacy in their relationships had led some to contemplate seeking fulfilment outside their partnerships. Furthermore, this lack of intimacy seemed to alter the dynamics of their relationships, with many describing a shift from romantic or intimate connections to bonds more akin to friendship or sibling-like relationships.

### Perceived understanding and lack of awareness

Participants’ experiences of menopause were shaped by varying levels of understanding and awareness. Attitudes towards treatment differed, with some sceptical of hormone therapy and others finding relief through local options. Limited guidance from healthcare providers often led women to seek information elsewhere, while greater knowledge and acceptance helped to support intimacy and mutual understanding in relationships.

#### Perceptions of menopausal treatment

The women expressed varied attitudes towards managing menopausal symptoms. While most had sought some form of relief, a clear divide emerged between those sceptical of hormone therapy and those open to alternative treatments. Several women maintained their scepticism regarding hormonal interventions. ‘*It’s just that hormones, I’m so afraid they’ll affect my mood and also my mental health with the hormones, I felt quite… I didn’t want to try it*’ (Participant 1).

The women who used local oestrogen treatment for dry mucous membranes found it to be an effective option. They reported that, after initiating local treatment, achieving orgasms became more attainable. The preference for local treatment was attributed to its perceived lower risk of side effects. However, some women had not sought medical care to explore potential treatment options.

#### Access to advice and information

The women expressed a strong desire to support others by sharing their experiences related to sexuality. They advocated for the establishment of specialised menopause clinics where individuals in their 50 s could receive guidance on managing sexual health, understanding symptoms, and knowing when to seek professional care.

They reported limited guidance from healthcare providers, and said that they often turned to informal sources such as peers, the media, and literature. While self-sought information improved understanding, some found the volume of available content overwhelming and confusing. In contrast, those with prior knowledge or greater acceptance of menopausal changes were less inclined to seek further information.

*I’ve taken advice from what I’ve been told. It’s been about talking about it, but the focus has been on the discomforts I experience, and it hasn’t been specifically about sexuality. Instead, the focus has been mainly on these other physical issues.* (Participant 7)

Participants further underscored the importance of developing a deeper understanding of the sexual changes occurring within themselves, as well as those experienced by men as they age, to enhance partner relationships and foster mutual support.

## Discussion

The findings of this study suggest that the menopausal transition is not experienced solely as a biological or physiological phenomenon, but rather as a multifaceted and deeply personal process that reshapes how women understand, navigate, and express their sexuality. Participants’ narratives demonstrate that sexual well-being is intricately entwined with broader psychosocial contexts, encompassing changes in relationship dynamics, shifts in self-perception and identity, and the development of emotional resilience.

The women in this study told us about various changes during menopausal transition that had affected their sex lives. von Hippel et al., [15] conducted a study in which they found that approximately 50% of women reported experiencing symptoms that significantly restricted their sexual activity during menopause. These symptoms, which can include vaginal dryness, hormonal fluctuations, and changes in sexual arousability, were identified as key factors influencing the sexual well-being of participants. These findings underscore the high prevalence of sexual dysfunction among menopausal women and indicate that menopause can have a profound impact on women’s sexual health and intimate relationships. Thomas et al. [16] identified several psychosocial factors contributing to a decline in sexual desire during menopause, including lifestyle-related stress, mood swings, and trauma. Our study also identified stress, in its various forms, as a key contributor to both diminished motivation to initiate sexual activity and a reduced frequency of desired sexual encounters.

Despite variations in sexual activity, participants consistently emphasised the importance of sexuality in their lives. Even those not currently engaged in sexual activity expressed a desire to rekindle their sex lives. During the interviews, many participants came to the realisation that they had rarely, if ever, spoken about sexuality with anyone. This lack of dialogue evoked a sense of sadness regarding the absence of sexual intimacy in their current lives.

Women who were not sexually active reported feelings of depression and an incomplete sense of self. These findings align with those of Gozuyesil et al. [6], who linked reduced sexual desire and absence of orgasm to lower quality of life, and Arcos-Romero and Calvillo [17], who connected female sexual health with psychological well-being. Participants emphasised that a fulfilling sex life encompasses more than penetrative sex, highlighting the significance of closeness, touch, interpersonal connection, and being seen and heard. Notably, even the participant who was single recognised these factors as vital in rekindling sexual desire.

A prominent theme in the findings was the impact of one’s partner’s reduced sexual desire during menopausal transition. As Rosen et al. [18] showed, diminished sexual function among men can negatively affect a couple’s sex life, with female partners reporting reduced relationship happiness and sexual satisfaction, although these effects are generally less pronounced in women than in men. Our results similarly indicate that partner-related sexual changes can undermine intimacy and contribute to relational strain. The combination of partners’ declining sexual interest and reluctance to discuss sexual changes or seek professional support often created emotional distance, making it difficult to maintain closeness. This dynamic amplified feelings of frustration, sadness, and sexual dissatisfaction, frequently placing the emotional burden of sustaining the sexual relationship on women. These findings are consistent with those of Bulut et al. [11], who found that women frequently expressed concern about their partners’ diminishing sexual engagement. The results highlight the relational nature of sexual well-being and the need to address both partners’ experiences in order to support intimacy.

Effective communication, both verbal and non-verbal, was identified as essential for maintaining a healthy sexual relationship; couples who openly addressed sexual challenges were more successful in resolving them, whereas avoidance often led to persistent difficulties. Morris [19] similarly underscores open communication as key to creating a supportive environment conducive to addressing sexual concerns within a relationship.

Raising awareness about sexuality during menopause is vital for promoting healthy ageing and improving women’s overall well-being. The findings of this study emphasise the importance of creating open dialogue and developing a nuanced understanding of the complex emotional, relational, and psychological changes that characterise menopausal transition. As Keye et al. [20] argue, educating women about the physical, emotional, and relational challenges associated with menopause is essential for empowering them to navigate these transitions effectively. Increased awareness and access to information can help mitigate negative outcomes such as sexual dysfunction and deterioration in the quality of intimate relationships.

The findings highlight the need for integrative, person-centred approaches to sexual health during menopausal transition. Sexual counselling and couple-centred strategies were described as particularly beneficial for addressing both partners’ sexual changes, enhancing communication, and maintaining emotional closeness [21], [22]. Accessible guidance and specialised menopause services could empower women and their partners to navigate sexual changes, mitigate dysfunction, and preserve relational quality. In addition to individual-focused interventions, there is growing recognition of the importance of adopting a couple-centred approach in addressing sexual health and well-being among midlife couples [22].

Rather than consulting doctors, many women rely on alternative sources of support during menopause, reflecting limited awareness of the healthcare options available to address their physical, sexual, and psychological needs [23]. This gap highlights the shortcomings of conventional biomedical approaches, which often fail to capture the complex, interrelated dimensions of women’s experiences during midlife [24]. Targeted interventions, such as sexual counselling, demonstrate the benefits of integrative, person-centred care and the importance of raising women’s awareness about the complex changes they experience during the menopausal transition [21].

## Strengths and limitations

The decision to employ a qualitative interview study design was informed by the objective of gaining a nuanced understanding of women’s experiences of menopause from their own perspectives. Women aged 50–60 years were selected as the target group because this age range represents a critical stage in the menopausal transition. Participants were recruited from diverse socio-economic backgrounds, allowing for a broad range of perspectives and contributing to the depth of the qualitative data. However, the relatively small sample size constitutes a limitation of the study. In addition, recruitment was conducted using a controlled snowball sampling approach intended to broaden the recruitment reach while limiting overrepresentation of closely connected social networks. Despite this strategy, snowball sampling is a non-probability sampling technique in which participants recruit others within their social networks. This means that not all members of the target population have an equal probability of selection [14]. Consequently, individuals within the same networks may share similar characteristics, and this may introduce selection bias and limit the representativeness of the sample. These potential limitations were considered when interpreting the study findings.

The presence of two researchers during interviews, as opposed to a traditional one-to-one format, may have influenced the dynamics of the conversation. This setup can affect the participant’s comfort level and openness, potentially reinforcing power imbalances. As such, the implications of this asymmetrical power relation should be acknowledged as a methodological limitation. To mitigate this, roles were clearly explained, with one researcher asking questions and the other taking notes, ensuring coverage of key topics while maintaining participant comfort. However, this approach may also be seen as a strength, because it allowed for richer observational insights, enhanced reflexivity, and facilitated prompt triangulation of interpretations during and immediately after the interviews. Thus, while the dual-interviewer format may have introduced certain constraints, it also contributed to the depth and rigour of the analytical process.

The authors acknowledge their extensive experience of working with women, which underscores the importance of critically reflecting upon potential biases and preconceptions during both the development of the interview guide and the analysis of the data. Reflexivity was further maintained throughout the analytical process via collaborative coding discussions, in which team members independently coded transcripts and then compared interpretations to identify and resolve discrepancies. These discussions allowed the team to interrogate assumptions, question potential biases, and ensure that interpretations remained grounded in the participants’ experiences rather than the researchers’ preconceptions.

To strengthen the study’s credibility, the authors undertook rigorous preparatory work and ensured active involvement by all members of the research team, who each contributed their specialised expertise. This collaborative approach helped to mitigate any biases and ensured a more comprehensive analysis. Throughout the process, the team maintained a reflexive stance, using discussion and triangulation to enhance trustworthiness and minimise interpretive bias.

The depth of the interviews, which averaged 45 min, was another strength, with participants providing significant and thoughtful contributions. Many unexpected insights emerged during the interviews, enriching the data in ways that exceeded initial expectations and providing a more complex understanding of the experiences being studied.

A strength of this qualitative study was the achievement of data saturation, evidenced by the absence of new codes, categories, or themes emerging from the final interviews [25]. As participant responses became repetitive and largely confirmatory, recruitment was concluded once no additional conceptual insights emerged, suggesting that the sample was sufficient to capture the core themes relevant to the research questions. However, the transferability of the findings to a broader context remains open to discussion. A potential limitation of the study is the imbalance between participants in relationships and those who were single, which may have influenced the range of experiences and perspectives shared, particularly in relation to sexual health and intimacy during menopause.

This discrepancy could limit the applicability of the findings to a wider population, especially one including people who may have different relationship dynamics. Therefore, while the study provides valuable and contextually rich insights, the findings should be viewed as specific to the participants and the setting in which the research was conducted and may not reflect the experiences of all individuals in similar situations.

For future research, we recommend conducting studies at multiple sites or across different countries to examine the influence of contextual and cultural factors on the phenomena under study. Additionally, exploring complementary methodological approaches, such as mixed methods or longitudinal designs, could further validate and extend the present findings, providing a more comprehensive understanding of participants’ experiences and perspectives.

## Conclusion

These results provide context-dependent insights rather than firm causal claims, emphasising that relationship dynamics interact with menopausal experiences in complex ways.

Emotional distance and poor communication with partners often reduce sexual desire more than menopausal symptoms themselves. Partners’ declining sexual interest and reluctance to discuss sexual changes can undermine intimacy, while limited awareness and lack of open discourse about sexuality leave women without adequate support. At the same time, the increased self-confidence and body acceptance that develop with age can enhance sexual fulfilment when relational support is present. These findings highlight the importance of addressing partner-related challenges, encouraging open communication, and providing tailored support. Participants also emphasised the need for healthcare services to provide accessible and comprehensive guidance that addresses both the sexual and relational aspects of well-being during the menopausal transition. This would help them to become more aware of and better prepared for the sexual changes that may occur during this stage of life.

## CRediT authorship contribution statement

**Anette Lycke:** Writing – review & editing, Writing – original draft, Validation, Methodology. **Karin Fristedt:** Writing – original draft, Visualization, Validation, Methodology, Investigation, Formal analysis, Data curation. **Ewa Andersson:** Writing – review & editing, Writing – original draft, Visualization, Validation, Supervision, Methodology, Investigation, Formal analysis, Data curation, Conceptualization. **My Högblom:** Writing – original draft, Visualization, Validation, Methodology, Investigation, Formal analysis, Data curation.

## Funding

There was no additional external funding received for this study.

## Declaration of Competing Interest

The authors declare that they have no known competing financial interests or personal relationships that could have appeared to influence the work reported in this paper.
